# Imipramine Influences Body Distribution of Supplemental Zinc Which May Enhance Antidepressant Action

**DOI:** 10.3390/nu12092529

**Published:** 2020-08-20

**Authors:** Anna Rafało-Ulińska, Ewa Poleszak, Aleksandra Szopa, Anna Serefko, Magdalena Rogowska, Ireneusz Sowa, Magdalena Wójciak, Bożena Muszyńska, Agata Krakowska, Joanna Gdula-Argasińska, Katarzyna Kała, Barbara Jasiewicz, Włodzimierz Opoka, Bernadeta Szewczyk, Gabriel Nowak

**Affiliations:** 1 Department of Neurobiology, Polish Academy of Sciences, Maj Institute of Pharmacology, 12 Smętna Street, 31-343 Kraków, Poland; rafalo@if-pan.krakow.pl (A.R.-U.); nowak@if-pan.krakow.pl (G.N.); 2 Department of Applied and Social Pharmacy, Laboratory of Preclinical Testing, Faculty of Pharmacy, Medical University of Lublin, Chodźki Street 1, 20-093 Lublin, Poland; ewa.poleszak@umlub.pl (E.P.); aleksandra.szopa@umlub.pl (A.S.); anna.serefko@umlub.pl (A.S.); 3 Department of Applied and Social Pharmacy, Faculty of Pharmacy, Medical University of Lublin, Chodźki Street 1, 20-093 Lublin, Poland; magda.nalesniak@umlub.pl; 4 Department of Analytical Chemistry, Faculty of Pharmacy, Medical University of Lublin, Chodźki street 4a, 20-093 Lublin, Poland; ireneusz.sowa@umlub.pl (I.S.); magdalena.wojciak-kosior@umlub.pl (M.W.); 5 Faculty of Pharmacy, Jagiellonian University Medical College, Medyczna street 9, 30-688 Krakow, Poland; bozena.muszynska@uj.edu.pl (B.M.); agata.krakowska@uj.edu.pl (A.K.); joanna.gdula-argasinska@uj.edu.pl (J.G.-A.); kasia.lisowska@doctoral.uj.edu.pl (K.K.); wlodzimierz.opoka@uj.edu.pl (W.O.);; 6 Department of Orthopedics and Rehabilitation, Jagiellonian University Medical College, Balzera street 15, 34-500 Zakopane, Poland; barbara.jasiewicz@uj.edu.pl

**Keywords:** imipramine, zinc, brain, blood, gastrointestinal tract, zinc transporters, permeability

## Abstract

Zinc (Zn) was found to enhance the antidepressant efficacy of imipramine (IMI) in human depression and animal tests/models of depression. However, the underlying mechanism for this effect remains unknown. We measured the effect of intragastric (*p.o.*) combined administration of IMI (60 mg/kg) and Zn (40 mg Zn/kg) in the forced swim test (FST) in mice. The effect of Zn + IMI on serum, brain, and intestinal Zn concentrations; Zn transporter (ZnT, ZIP) protein levels in the intestine and ZnT in the brain; including BDNF (brain-derived neurotrophic factor) and CREB (cAMP response element-binding protein) protein levels in the brain were evaluated. Finally, the effect of IMI on Zn permeability was measured in vitro in colon epithelial Caco-2 cells. The co-administration of IMI and Zn induced antidepressant-like activity in the FST in mice compared to controls and Zn or IMI given alone. This effect correlated with increased BDNF and the ratio of pCREB/CREB protein levels in the prefrontal cortex (PFC) compared to the control group. Zn + IMI co-treatment increased Zn concentrations in the serum and brain compared to the control group. However, in serum, co-administration of IMI and Zn decreased Zn concentration compared to Zn alone treatment. Also, there was a reduction in the Zn-induced enhancement of ZnT1 protein level in the small intestine. Zn + IMI also induced an increase in the ZnT4 protein level in the PFC compared to the control group and normalized the Zn-induced decrease in the ZnT1 protein level in the hippocampus (Hp). The in vitro studies revealed enhanced Zn permeability (observed as the increased transfer of Zn through the intestinal cell membrane) after IMI treatment. Our data indicate that IMI enhances Zn transfer through the intestinal tract and influences the redistribution of Zn between the blood and brain. These mechanisms might explain the enhanced antidepressant efficacy of combined IMI/Zn treatment observed in the FST in mice.

## 1. Introduction

The efficacy of current antidepressant therapy is not satisfactory, resulting in the introduction of poly/adjunctive agents to enhance the activity of classical agents in the treatment of depression. Recently, Nowak et al. (2003) [1] demonstrated the potency of zinc (Zn) as an adjunct to tricyclic antidepressants and selective serotonin reuptake inhibitors (SSRIs) in the treatment of unipolar depression. Zn supplementation has been shown to augment the efficacy and speed of the onset of therapeutic response to IMI treatment, especially in treatment-resistant depression [2]. Ranjbar et al., [3] confirmed the beneficial effects of Zn supplementation in conjunction with SSRI antidepressant drugs. More so, Zn is known to be efficacious as a monotherapy for the treatment of depressive symptoms in obese patients [4]. Preclinical studies have equally demonstrated the antidepressant-like activity of Zn as well as the enhancement of antidepressant efficacy after co-treatment with Zn and antidepressant drugs in various tests and models of depression [5,6]. Much research has been devoted to study the effects of the joint administration of Zn + IMI in animal models of depression. Different dose regimens of Zn + IMI treatment are active in the FST in mice and rats following intraperitoneal (*i.p*.) administration [7,8,9]. Such effects have also been demonstrated in the tail suspension test (TST) after per os (*p.o.*) administration [10]. Both acute and chronic treatments with Zn + IMI reversed the inhibitory activity of chronic dexamethasone treatment (Wrobel et al., 2015) and also reversed the effects of chronic restraint stress (CRS) in mice [11,12]. The mechanism underlying the beneficial effects of co-treatment with Zn seems to be associated with the antagonism of N-methyl-D-aspartate (NMDA) glutamatergic receptors, modulation of α-amino-3-hydroxy-5-methyl-4-isoxazole propionic acid (AMPA) receptors, and interaction with 5-HT1A serotonin receptors [5,13,14]. Recent studies suggest that the mechanism of the antidepressant activity of Zn + IMI treatment is related to the enhanced phosphorylation of the mammalian target of rapamycin (mTOR) kinase and mRNA expression of cAMP response element binding protein (CREB) and brain derived neurotrophic factor (BDNF) [7,12]. Also, the administration of Zn + IMI normalizes the CRS-induced changes in Zn serum and brain (hippocampus) levels [12]. On the other hand, chronic treatment with IMI in naïve rats leads to increased hippocampal concentration of Zn [15].

Based on the preceding data, the present study was designed to further evaluate the mechanisms responsible for the behavioral effects of Zn + IMI treatment in the context of Zn homeostasis, absorption, and redistribution.

## 2. Material and Methods

### 2.1. Animals

All experiments were carried out on naïve adult male Albino Swiss mice (25–30 g) purchased from a licensed breeder (Kołacz, Warsaw, Poland). The animals were housed in environmentally controlled rooms with a 12 h day/night cycle, in standard cages under strictly controlled laboratory conditions (temperature maintained at 22 ± 2 °C with relative humidity of 40–60%). Throughout the study, the animals were given *ad libitum* access to water and food (Altromin, Lage, Germany; Zn ~95 mg/kg). Animals were acclimated to laboratory conditions for a period of at least one week before experiments began. Experiments were conducted between 8:00 a.m. and 3.00 p.m. to minimize circadian influences. Procedures involving mice and their carein the present study were approved by the Local Ethics Committee at the Medical University of Lublin (license no 42/2011) and were performed in accordance with European standards related to experimental studies on animal models. Experimenters were blinded to the treatment conditions.

### 2.2. Drug Administration

Imipramine hydrochloride (IMI, 60 mg/kg, Sigma-Aldrich, Poznań, Poland) and zinc hydroaspartate (40 mg/kg refer to pure zinc ions, Farmapol, Poznań, Poland) were dissolved in 0.9% NaCl just before the start of experimentation. Doses of IMI and zinc (Zn) were selected based on results of our preliminary studies. The respective agents were administered per os (*p.o.*) 60 min before behavioural testing using the following scheme: (I group) 0.9% NaCl + 0.9% NaCl (control group); (II group) Zn (40 mg/kg) + 0.9% NaCl; (III group) IMI (60 mg/kg) + 0.9% NaCl; (IV group) Zn (40 mg/kg) + IMI (60 mg/kg). The volume of vehicle or drug solution for *p.o.* administration was 5 mL/kg.

### 2.3. Forced Swim Test (FST)

The procedure was carried out according to the method of Porsolt et al. [16]. Mice were individually placed into glass cylinders (height 25 cm, diameter 10 cm) containing 10 cm of water at 23–25 °C. The total duration of immobility was recordedduring the last 4 min of the 6-min long testing period. A mouse was judged immobile when it ceased struggling and remained floating motionless in the water. The results obtained in the FST are expressed as the arithmetic mean of immobility time of animals given in seconds ± standard error of the mean (S.E.M) for each experimental group.

### 2.4. Locomotor Activity

Locomotor activity was measured using Opto-Varimex-4 Auto-Track (Columbus Instruments, Columbus, OH, USA). The actometer consists of four transparent cages with a lid (43 × 43 × 32 cm), a set of four infrared emitters (each emitter has 16 laser beams), and four detectors which monitor animal movements. Mice were placed individually in the cages for 6 min. Locomotor activity was evaluated between the 2nd and the 6th minute, which corresponded with the time interval analyzed in the FST. The results obtained in the locomotor activity test are presented as the arithmetic average distance that a mouse travelled (in cm) ± S.E.M for each experimental group.

### 2.5. Determination of Zn (II) in Serum and Brain Homogenates

Mice were decapitated sixty minutes following drug administration. Blood was collected into Eppendorf tubes and allowed to clot at room temperature. Subsequently, the blood was centrifuged at 10,000 rpm for 10 min and serum collected into polyethylene tubes and frozen at −25 °C. Immediately after decapitation, brains were dissected from the skull, washed with 0.9% NaCl and also frozen at −25 °C.

Serum and brain concentrations of IMI/Zn were assayed using a high-resolution continuum source atomic absorption spectrometer ContrAA 700 (Analytik Jena, Jena, Germany) with a 300 W xenon short-arc lamp as a continuum radiation source and a CCD array detector with a resolution of 2 pm per pixel in the far-ultraviolet range. The instrument was operated using Aspect CS 2.0.0 software (Analytik Jena). The optimization of the method was performed experimentally using a certified material Seronorm Trace Elements Whole Blood L-1. The measurement was conducted at λ = 213,857 nm in flame mode (gas flow 50 L/h). The calibration curve in the range of 0–20 mg/L was prepared from a stock solution of 1000 mg/L Zn (CertiPUR^®^, Merck, Darmstadt, Germany). The validation parameters (n = 5) were as follow: calibration curve y= (−0.0001968 + 0.0580274)/(1 + 0.0201765x), correlation coefficient R = 0.9999, characteristic concentration 0.04513 mg/L (1% of absorbance), precision 0.7–2.9% RSD.

Tissue and blood samples were digested with a mixture of HCl and HNO_3_ (3:1, *v*/*v*) (Suprapur^®^, Merck, Darmstadt, Germany) and deionized water (2:3, *v*/*v*) using a microwave digestion system (Novawave^®^, SCP Science, Baie-D’Urfe, QC, Canada) for 30 min at 200 °C.

### 2.6. Determination of Zn Transport Using Caco-2 Cells

Colon epithelial Caco-2 cells (HTB-37) were cultured in EMEM medium with 15% Fetal Bovine Serum, penicillin (100 IU/mL), and streptomycin (100 µg/mL) (ATCC, Manassas, VA, USA) at 37 °C in a humidified atmosphere containing 5% CO_2_. Caco-2 cells were seeded onto PET membranes (0.4 μm pore size) inside an SPL Life Sciences 6-well Insert system (SPL Life Sciences, Pocheon-si, Korea) at a density of 2 × 10^4^ cells/well and grown for 21 days. The integrity of the colon epithelial cell monolayer was determined by measuring the trans-epithelial electrical resistance (TEER) using a Millicell ERS-2 Epithelial Volt-Ohm Meter electrical resistance system (Millipore, Merck, Darmstadt, Germany). Cells with TEER values above 800 Ω × cm^2^, indicating tightness of the junctions between intestinal epithelial cells, were used in the experiments. Solutions containing Zn ions (27.2 mg/L) or imipramine hydrochloride (120 mg/L) with Zn (27.2 mg/L) were applied to the surface of the monocyclic Caco-2 cells. Each variant was incubated for 30 and 90 min at 37 °C. Zn (F-AAS) and IMI (RP-HPLC) were sampled from above and below the monolayers of the Caco-2 cells.

### 2.7. Zn (II) Analysis by F-AAS Method

The concentration of Zn ions was determined in the samples by atomic absorption spectrometry using an ICE 3000 Series (Thermo Fisher Scientific, Leicestershire, UK) with a flame atomizer (F-AAS). The least-squares calibration curve was determined for a concentration range of 0.1–1 μg/g. Samples were analyzed at a wavelength of 217.0 nm and a gap width of 0.5 nm. Suction time and measurement time was 3 s each time. Acetylene was used as a flammable gas and oxidizer.

### 2.8. Western Blot Analysis

Samples were homogenized on ice in a 2% solution of sodium dodecyl sulfate (SDS), following which each homogenate was denatured at 95 °C for 10 min and finally centrifuged for 5 min at 10,000 rpm at 4 °C. After centrifugation, the supernatant was collected, and the protein content determined using the bicinchoninic acid assay (Pierce Biotechnology, Inc., Rockford, IL, USA). Samples containing 15 µg of protein were mixed with sample buffer (Invitrogen, Paisley, UK) andfractionated on a 10.0% SDS-polyacrylamide gel and transferred to a nitrocellulose membrane (Invitrogen, Paisley, UK). To block non-specific binding, 1% blocking solution was used (BM Chemiluminescence Western Blotting Kit (Mouse/Rabbit), Roche, Switzerland). After blocking, membranes were incubated overnight at 4 °C with the respective antibodies: rabbit polyclonal anti-ZnT1 antibody (1:1000; Synaptic System, Goettingen, Germany), mouse monoclonal anti-ZnT3 antibody (1:1000; Synaptic System), rabbit polyclonal anti-ZnT4 antibody (1:1000; GeneTex, Irvine, CA, USA); goat polyclonal anti-ZnT5 antibody (1:1000, Santa Cruz Biotechnology, Dallas, TX, USA); goat polyclonal anti-ZnT6 antibody (1:1000, Santa Cruz); rabbit polyclonal anti-ZIP1 antibody (1:1000, GeneTex); rabbit polyclonalanti-ZIP4 antibody (1:1000, Abcam, Cambridge, UK); rabbit polyclonal anti-ZIP5 antibody (1:1000, Sigma-Aldrich); rabbit polyclonal anti-ZIP12 antibody (1:1000, Sigma-Aldrich); mouse monoclonal anti-DMT1 antibody (1:1000, Abcam); rabbit polyclonal anti-BDNF antibody (1:200, Santa Cruz Biotechnology); mouse monoclonal anti-CREB antibody (1:1000, Millipore); rabbit polyclonal anti-phospho CREB antibody 1:1000, Millipore). All antibodies were dissolved in 0.5% blocking reagent (Roche). The next day, the membranes were washed three times for 10 min in Tris-buffered saline with Tween (TBS-T) and incubated for 60 min with an anti-mouse IgG-peroxidase conjugated/anti-rabbit IgG-peroxidase conjugated antibody (1:7000). This set of secondary antibodies was also a component of the BM Chemiluminescence Western Blotting Kit (Mouse/Rabbit). After incubation, the membranes were washed three times for 10 min with TBS-T. In the last step, the blots were incubated with a detection reagent (Roche). The signal from the tested proteins was visualized and measured using a Fuji-Las 1000 system and Fuji Image Gauge v.4.0 software. To check for transfer and loading, β-actin was indicated on each blot. For this, a goat anti-β-actin antibody (1:5000, Abcam) was used. Further procedures were the same as for the other proteins. The final result is presented as the ratio of the optical density of the protein to the optical density of β-actin. For CREB protein additionally p-CREB/CREB ratio was analyzed.

### 2.9. Statistical Analysis

The results of the study were analyzed by one-way or two-way analysis of variance (ANOVA) with the Tukey multiple comparison post-hoc test and Student’s *t*-test when appropriate. All statistical analyses were performed using GraphPad (Prism). The results are presented as means ± S.E.M. A *p* < 0.05 was considered a statistically significant difference.

## 3. Results

### 3.1. The Effect of Coadministration of Zn and IMI in the FST in Mice

**Figure 1:**
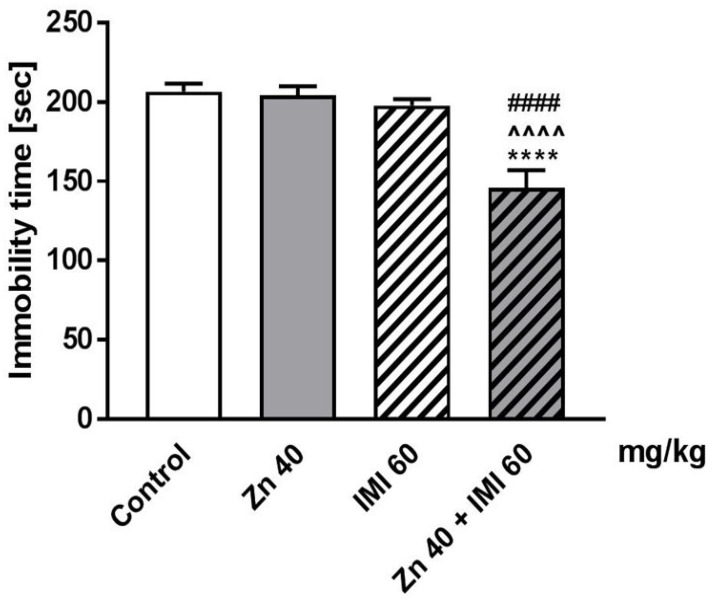
Effect of combined treatment of Zn and IMI in the FST in mice. Zn (40 mg Zn/kg), IMI (60 mg/kg), and NaCl (control) were administered *p.o.* 60 min before the test. Results are expressed as mean ± S.E.M (n = 9–10 per group). **** *p* < 0.0001 vs. control group, ^^^^ *p* < 0.0001 vs. IMI treated group, ^####^ *p* < 0.0001 vs. Zn treated group (two-way ANOVA followed by Tukey *post-hoc* test).

The effect of the co-administration of Zn and IMI on the total duration of immobility time in mice is shown in Figure 1. Zn (40 mg Zn/kg) administered in combination with IMI (60 mg/kg) significantly reduced the immobility time in the FST in mice (*p* < 0.0001) compared to the control group. On the other hand, treatment with Zn or IMI alone did not induce any significant changes in the total duration of immobility time in the FST compared to the control group (*p* > 0.05).

Two-way ANOVA demonstrated a significant effect of IMI [F(1,34) = 14.35, *p* = 0.0006], a significant effect of Zn [F(1,34) = 22.14, *p* < 0.0001], and significant interaction [F(1,34) = 12.05, *p* = 0.0014].

### 3.2. The Effect of Coadministration of Zn and IMIon Locomotor Activity of Mice

The effect of co-administration of Zn and IMI on spontaneous locomotor activity of mice is shown in Table 1. Zn or IMI administered either alone or in combination had no statistically significant effect on the locomotor activity of mice (*p* > 0.05).

Two-way ANOVA demonstrated no effect of IMI [F(1,42) = 2.053, *p* = 0.1593], no effect of Zn [F(1,42) = 2.785, *p* = 0.1026], and no interaction [F(1,42) = 0.005, *p* = 0.9422].

### 3.3. The Effect of Zn and IMI on the BDNF and pCREB/CREB Protein Level in the PFC and Hp of Mice Brain

**Figure 2:**
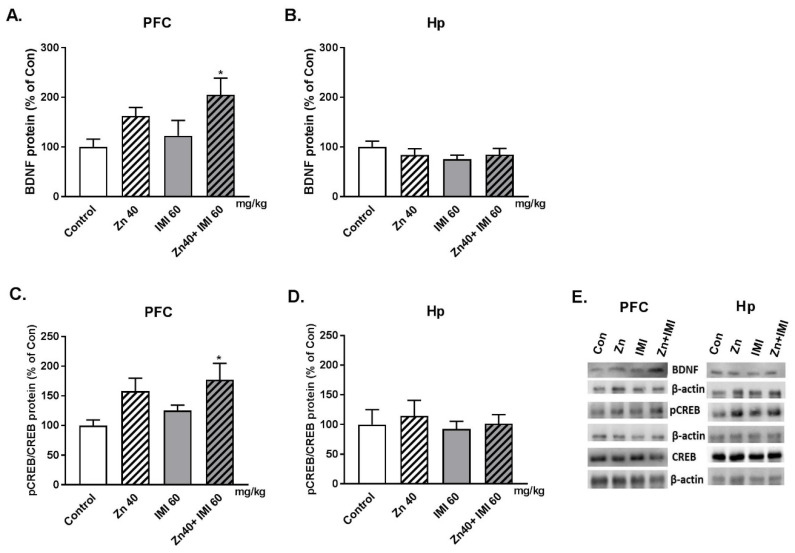
Western blot analysis of the effect of Zn (40 mg Zn/kg) + IMI (60 mg/kg), and NaCl (control) administered *p.o.* on the protein levels of BDNF (**A**,**B**) and p-CREB/CREB (**C**,**D**) in the PFC and Hp of mouse. Results are expressed as mean ± S.E.M (n = 5–7 per group). * *p* < 0.05 vs. control group (two-way ANOVA followed by Tukey *post-hoc* test) (**E**).

The effect of Zn and IMI on the BDNF and pCREB/CREB ratio protein level in the PFC and Hp of mice brain is shown in Figure 2. The co-administration of Zn and IMI induced statistically significant increase in the BDNF protein level. Trend to increase in BDNF was also observed after Zn alone treatment in PFC (Figure 2A). No changes in the BDNF protein level was found in the Hp (Figure 2B). Zn + IMI induced also increase in the p-CREB/CREB protein level in the PFC. Slight increase in the p-CREB/CREB ratio in PFC of mice treated with Zn and IMI alone (Figure 2C). Similar to BDNF, no changes in the p-CREB/CREB ratio was observed in the Hp of mice in all studied groups (Figure 2D). Two way ANOVA demonstrated for: BDNF in PFC—significant effect of IMI [F(1,17) = 8.205, *p* = 0.0107]; no effect of Zn [F(1,17)= 1.67; *p* = 0.2135]; no interaction [F(1,17) = 0.155; *p* = 0.6985]; BDNF in Hp—no effect of IMI [F(1,18) = 0.083, *p* = 0.7767]; no effect of Zn [F(1,18) = 1.208; *p* = 0.2862]; no interaction [F(1,18) = 1.27; *p* = 0.2745]; pCREB/CREB in PFC—significant effect of IMI [F(1,18) = 10, *p* = 0.0054]; no effect of Zn [F(1,18) = 1.624; *p* = 0.2187]; no interaction [F(1,18) = 0.0316; *p* = 0.8609]; pCREB/CREB in Hp—no effect of IMI [F(1,16) = 0.3196, *p* = 0.5797]; no effect of Zn [F(1,16) = 0.254; *p* = 0.6211]; no interaction [F(1,16) = 0.018; *p* = 0.8936].

### 3.4. The Effect of Zn and IMI on the Concentrations of Zn in Mouse Serum and Brain

The effect of Zn and IMI on serum and brain concentrations of Zn in mice is shown in Table 2. Zn (A) or IMI (B) administered alone increased serum Zn (*p* < 0.01 and *p* < 0.05, respectively). The co-administration of Zn and IMI (A) induced an increase in the concentration of serum and brain Zn (*p* < 0.001) compared to the control group. However, in the group that received Zn alone, there was a significant reduction in serum Zn (*p* < 0.01) and an increase in brain zinc (*p* < 0.01).

One-way ANOVA followed by the Tukey multiple comparison post-hoc test revealed the statistically significant differences in serum Zn concentration [F(2,27) = 29.91, *p* < 0.0001] and also brain Zn concentration [F(2,28) = 25.41, *p* < 0.0001].

### 3.5. The Effect of Zn + IMI on Zn Transport in Human Colon Epithelial Cells (In Vitro Studies)

**Figure 3:**
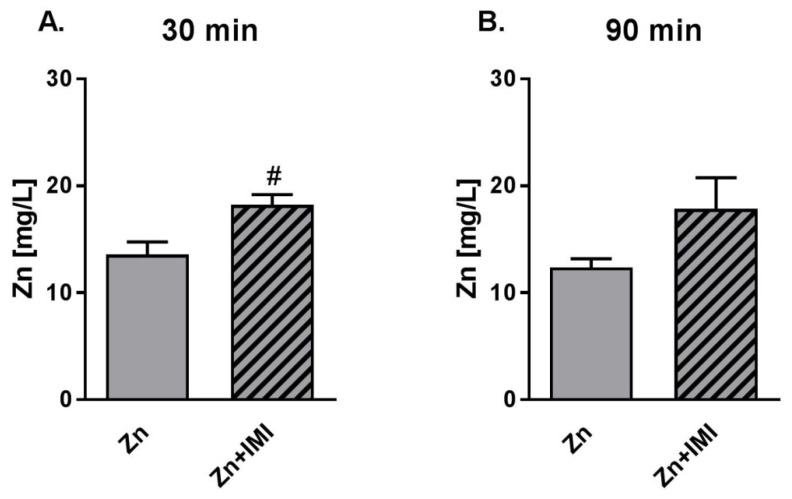
Effect of IMI on Zn transport in colon epithelial Caco-2 cells (HTB-37) 30 min (**A**) and 90 min (**B**) after the treatment. Zn (F-AAS) was sampled from below the monolayers of the Caco-2 cells. Results are expressed as mean ± S.E.M, n = 3. ^#^ *p* < 0.05 vs. Zn group (*t*-test).

The results obtained for Zn transport in human colon epithelial cells—Caco-2 (Figure 3) indicated that Zn was effectively transported, and that the co-administration of Zn (27.2 mg/L) and IMI (120 mg/L) enhanced transport of Zn 30 min after treatment (Figure 3A, *p* < 0.05). Slight increase in the Zn transport was also observed 90 min after Zn + IMI treatment (Figure 3B).

### 3.6. The Effect of Zn + IMI on the ZnT Transporters Protein Level in the Small Intestine

**Figure 4:**
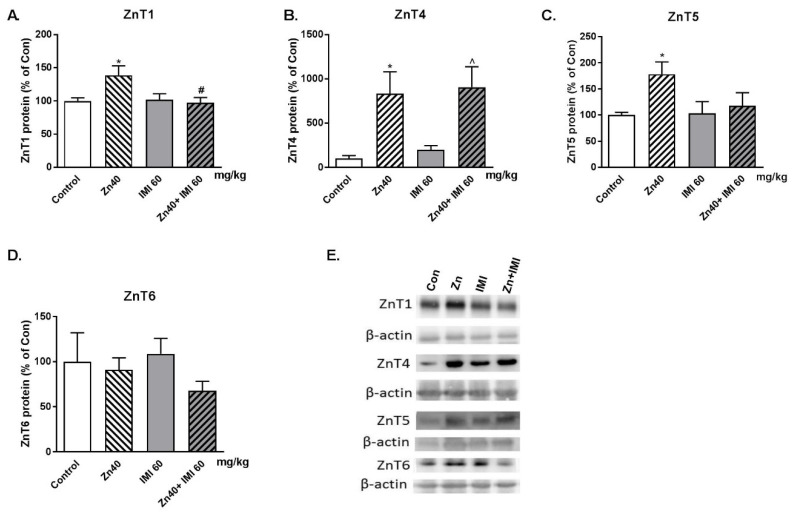
Western blot analysis of the effect of co-treatment of Zn (40 mg Zn/kg), IMI (60 mg/kg), and NaCl (control) administered *p.o.* on the protein levels of Zn transporters: (**A**) ZnT1, (**B**) ZnT4, (**C**) ZnT5, and (**D**) ZnT6 in the mouse small intestine. Representative western blots (**E**). Results are expressed as mean ± S.E.M (n = 5–7 per group). * *p* < 0.05 vs. control group, ^#^ *p* < 0.05 vs. Zn group, ^ *p* < 0.05 vs. IMI group (two-way ANOVA followed by Tukey *post-hoc* test).

The effect of Zn, IMI, and Zn + IMI treatment on the levels of ZnT1, ZnT4, ZnT5 and ZnT6 in the small intestine is shown in Figure 4A–D. Zn given alone induced statistically significant increases in ZnT1, ZnT4, and ZnT5 protein levels in the small intestine. Co-treatment with Zn + IMI normalized the Zn-induced increase in ZnT1 (*p* < 0.05) but not ZnT4 and ZnT5 protein levels. The representative western blots are shown in Figure 4E Two-way ANOVA for ZnT1 demonstrated: no effect of IMI [F(1,19) = 3.462, *p* = 0.0783], significant effect of Zn [F(1,19) = 4.704, *p* = 0.0430]; significant interaction [F(1,19) = 5.702, *p* = 0.0275]; ZnT4: significant effect of IMI [F(1,20) = 17.46, *p* = 0.0005], no effect of Zn [F(1,20) = 0,2526, *p* = 0.6207], no interaction [F(1,20) = 0.0039, *p* = 0.9507]; for ZnT5: significant effect of IMI [F(1,22) = 5.248, *p* = 0.0319], no effect of Zn [F(1,22) = 1.972, *p* = 0.1742], no interaction [F(1,22) = 2.481, *p* = 0.1295]; for ZnT6: no effect of IMI [F(1,19) = 1.803 = 0.1952], no effect of Zn [F(1,19) = 0.1472, *p* = 0.7054], no interaction [F(1,19) = 0.7339, *p* = 0.4023].

### 3.7. The Effect of Zn + IMI on ZIP Transporters and DMT1 Protein Levels in the Small Intestine

**Figure 5:**
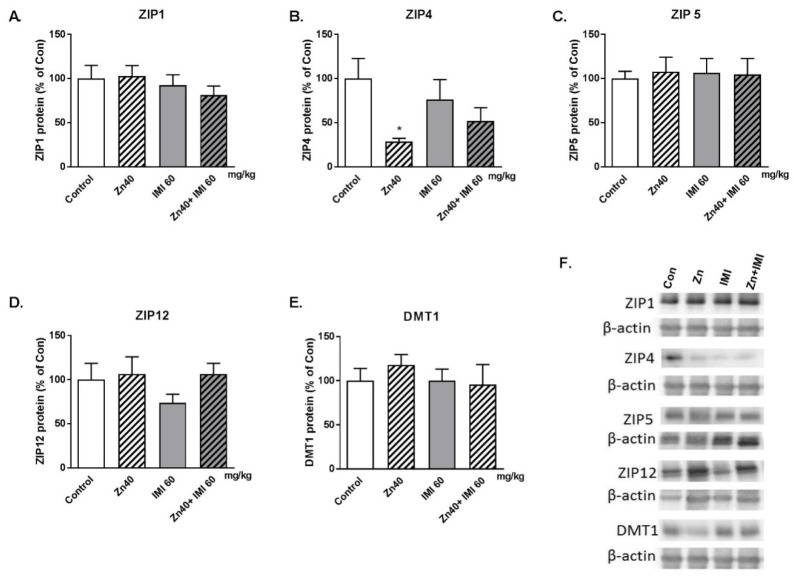
Western blot analysis of the effect of Zn (40 mg Zn/kg) + IMI (60 mg/kg), and NaCl (control) administered *p.o.* on the protein levels of zinc transporters: (**A**) ZIP1, (**B**) ZIP4, (**C**) ZIP5, (**D**) ZIP12, and (**E**) DMT1 in the mouse small intestine. Representative western blots (**F**). Results are expressed as mean ± S.E.M (n = 5–7 per group). * *p* <0.05 vs. control group (two-way ANOVA followed by Tukey *post-hoc* test).

The effect of Zn, IMI, and Zn + IMI treatment on the levels of ZIP1, ZIP4, ZIP5, ZIP12, and DMT1 in the small intestine is shown in Figure 5A–E. IMI given alone or in combination with Zn did not alter the levels of ZIP and DMT1 transporter protein levels in the small intestine. However, Zn given alone induced a decrease in ZIP4 protein level (Figure 5B). The representative western blots are shown in Figure 5F. Two-way ANOVA for ZIP1 demonstrated: no effect of IMI [F(1,19) = 0.104, *p* = 0.7506], no effect of Zn [F(1,19) = 1.321, *p* = 0.2647]; no effect [F(1,19) = 0.3055, *p* = 0.5869]; for ZIP4: significant effect of IMI [F(1,22) = 7.469, *p* = 0.0121], no effect of Zn [F(1,22) = 5.552 × 10^−7^, *p* = 0.9994], no interaction [F(1,22) = 1.779, *p* = 0.1960]; for ZIP5: no effect of IMI [F(1,24) = 0.036, *p* = 0.851], no effect of Zn [F(1,24) = 0.0123, *p* = 0.9123], no interaction [F(1,24) = 0.1029, *p* = 0.7511]; for ZIP12: no effect of IMI [F(1,23) = 1,65, *p* = 0.2117], no effect of Zn [F(1,23), *p* = 0.3944], no interaction [F(1,23) = 0.7442, *p* = 0.3972]; for DMT1: no effect of IMI [F(1,24) = 0.1714, *p* = 0.6825], no effect of Zn [F(1,24) = 0.4871, *p* = 0.4919], no interaction [F(1,24) = 0.4973, *p* = 0.4875].

### 3.8. The Effect of Zn and IMI on Zn Concentration in the Mouse Small Intestine

**Figure 6:**
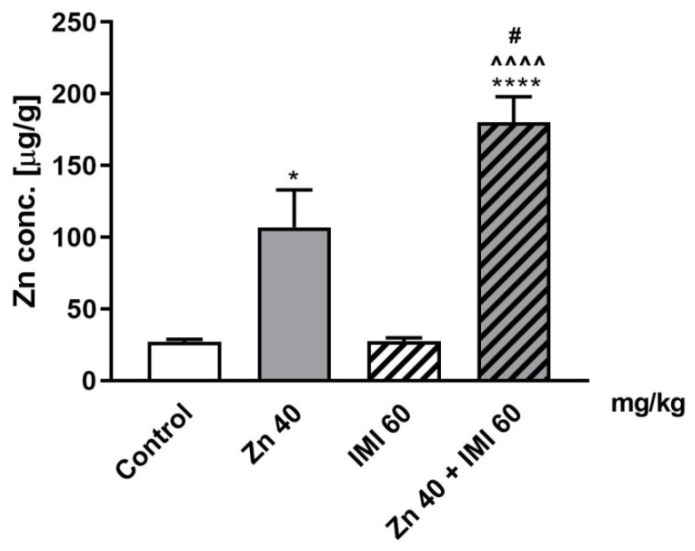
Effect of Zn (40 mg Zn/kg) + IMI (60 mg/kg), and NaCl (control) administered *p.o.* on Zn concentration in the mouse small intestine. Results are expressed as mean ± S.E.M (n = 4 per group). * *p* < 0.05; **** *p* < 0.0001 vs. control group, ^^^^ *p* < 0.0001 vs. IMI treated group, ^#^ *p* < 0.05 vs. Zn treated group (two-way ANOVA followed by Tukey *post-hoc* test).

The effect of Zn, IMI, and Zn + IMI on Zn concentration in the small intestine is shown in Figure 6. Zn given alone induced a statistically significant increase in Zn concentration in the small intestine (*p* < 0.05) and IMI treatment further enhanced this effect (*p* < 0.0001).

Two-way ANOVA demonstrated significant effect of IMI [F(1,12) = 53.72, *p* < 0.0001], significant effect of Zn [F(1,12) = 5.347, *p* = 0.0393], and significant interaction [F(1,12) = 5,24, *p* = 0.0410].

### 3.9. The Effect of Co-Administration of Zn and IMI on Protein Levels of Zn Transporters (ZnT1, ZnT3, and ZnT4) in the Mouse Brain

**Figure 7:**
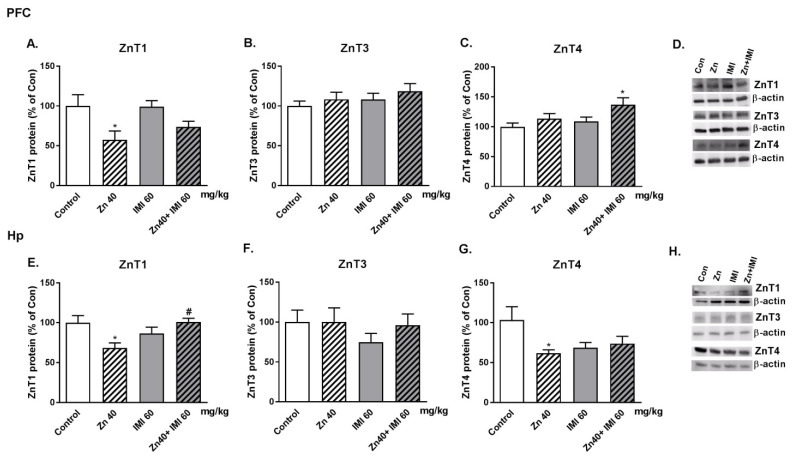
Western blot analysis of the effect of Zn (40 mg Zn/kg) + IMI (60 mg/kg), and NaCl (control) administered *p.o.* on the protein levels of Zn transporters: (**A**,**E**) ZnT1, (**B**,**F**) ZnT3, and (**C**,**G**) ZnT4 in the PFC and Hp of mouse brain. Representative western blots (**D**,**H**). Results are expressed as mean ± S.E.M (n = 5–6 per group). * *p* < 0.05 vs. control group, ^#^ *p* < 0.05 vs. Zn group (two-way ANOVA followed by Tukey *post-hoc* test).

The effect of Zn, IMI, and Zn + IMI treatment on the levels of ZnT1, ZnT3, and ZnT4 proteins in the PFC and Hp of the mouse brain is shown in Figure 7A–H. Zn given alone induced a statistically significant decrease in ZnT1 protein levels both in the PFC and Hp (*p* < 0.05) and ZnT4 protein level in the Hp (*p* < 0.05). No significant alterations in protein levels were observed after IMI treatment. Co-treatment with Zn and IMI reversed the Zn induced decrease in ZnT1 in the Hp but not in the PFC. Co-administration of Zn and IMI induced an increase in ZnT4 only in the PFC (*p* < 0.05). The representative western blots are shown in Figure 7D,H. Two-way ANOVA in the PFC for ZnT1 demonstrated: significant effect of IMI [F(1,16) = 10,63, *p* = 0.0049], no effect of Zn [F(1,16) = 0.5428, *p* = 0.4720], no interaction [F(1,16) = 0.6808, *p* = 0.4214]; for ZnT3: no effect of IMI [F(1,20) = 1.295, *p* = 0.2685], no effect of Zn [F(1,20) = 1.258, *p* = 0.2753], no interaction [F(1,20) = 0.0146, *p* = 0.9048]; for ZnT4: significant effect of IMI [F(1,19) = 6.185, *p* = 0.0223], no effect of Zn [F(1,19) = 3.72, *p* = 0.0688], no interaction [F(1,20) = 0.720, *p* = 0.4067]; in the Hp: for ZnT1: no effect of IMI [F(1,17) = 1.475, *p* = 0.2411], no effect of Zn [F(1,17) = 0.1830, *p* = 0.1938], significant interaction [F(1,17) = 10,50, *p* = 0.0048]; for ZnT3: No effect of IMI [F(1,18) = 0.490, *p* = 0.4925], no effect of Zn [F(1,18) = 0.932, *p* = 0.3470], no interaction [F(1,18) = 0.4906, *p* = 0.4926]; for ZnT4: no effect of IMI [F(1,20) = 3.117, *p* = 0.0927], no effect of Zn [F(1,20) = 1.207, *p* = 0.2851], no interaction [F(1,20) = 5.187, *p* = 0.0339].

## 4. Discussion

Clinical studies have demonstrated that Zn adjuvant therapy can improve the antidepressant activity of IMI in patients with major depression [1,2]. Preclinical studies have also shown the antidepressant-like effect of Zn + IMI in different tests and models of depression, further confirming that Zn supplementation might lower the effective dose of IMI, leading to limited side effects induced by IMI treatment [7,9,10,12,17,18].

In the present study Zn + IMI induced a significant antidepressant-like effect in the FST in mice. The novelty of this work is the fact that the Zn and IMI were administered *p.o*. Furthermore, we used a dose higher than before −40 mg Zn/kg body weight (zinc hydroaspartate). This dose was established in our preliminary data studying dose response effect of Zn administered *p.o.* (data not shown). The dose (40 mg Zn/kg, *p.o*.) was not effective in FST (when administered without IMI), while for example, in our previous study in mice the dose of 30 mg Zn/kg (but not 15 mg Zn/kg) of Zn administered *i.p*. was effective [11].

In humans, the recommended dietary allowance (RDA) for Zn is 8–11 mg/day in women and men respectively [19]. However, the LD_50_ value for humans is estimated as 27 g Zn/day [20]. The doses effective in animals are different than those for humans. For example for mice LD_50_ after oral administration varied between 86–605 mg Zn/kg depending of salt used [21].

This behavioral effect of Zn + IMI observed in the FST in mice was accompanied by an increase in BDNF and the ratio of pCREB/CREB proteins in the PFC, which is a common feature of antidepressant drug action [22]. However, we did not observe changes in BDNF or CREB protein levels in the Hp of mice. This effect differs from that observed by Ding et al. [12], or Huang et al. [7], who found an increase in mRNA and protein expression of BDNF and CREB in the Hp of mice and rats respectively [7,12]. This difference may, however, result from: different routes of administration (intragastric vs. intraperitoneally), doses of both Zn and IMI, and protocols of behavioral studies (naive vs. stressed animals) [7,12]. The other mechanism underlying the antidepressant activity of Zn and IMI may be related to the activation of the mTOR signaling pathways [7]. There are currently no studies that have evaluated the possible changes in the absorption, distribution, and homeostasis of Zn following Zn + IMI co–treatment.

Zn homeostasis is controlled by a several proteins, including Zn transporters (ZIP and ZnT family of proteins). ZIP and ZnT group of proteins perform opposing functions. ZIP transporters (14 members) transport Zn^2+^ from the extracellular space or from intracellular vesicles to the cytoplasm while ZnTs (10 members) mediate membrane Zn^2+^ transport and regulate intracellular and cytoplasmic Zn^2+^ levels [23,24,25,26]. Given the function and location of these transporters (intestine, brain) and their possible involvement in the etiology of diseases [23,24,25,26], especially mental diseases [26,27,28,29], our study focused on ZnT1, ZnT3, ZnT4, ZnT5, ZnT6, and ZIP1, ZIP4, ZIP5, ZIP12 proteins and their role in the mechanism of IMI/Zn action.

ZnT1 is expressed in the plasma membrane and functions to export Zn^2+^ from the cytosol to the extracellular space during periods of elevated levels of cytosolic Zn^2+^. ZnT3 is specific to the brain and mainly localized on synaptic vesicles. ZnT4 is found on the plasma membrane of the cell and in endosomal/secretory vesicles. ZnT3 and ZnT4 are involved in the transport of Zn to cellular compartments. ZnT5 and ZnT6 are localized on the membrane of the Golgi apparatus and cytoplasmic vesicles. ZnT5-ZnT6 heterodimers carry out essential biosynthetic functions by delivering Zn into the early secretory pathway, where it is required for the activation of zinc-dependent enzymes [24,25]. ZIP1 functions as an importer of Zn and is expressed on the plasma membrane. ZIP4 is essential in dietary Zn absorption in mammals and highly expressed in the small intestine; its expression is strictly regulated by Zn levels. ZIP5 functions as a Zn importer. It is expressed in the small intestine and pancreas. Its expression is correlated with Zn availability and decreased in zinc-depleted environments. ZIP6 is localized to the plasma membrane and acts as a Zn transporter. ZIP12 is also expressed in the membrane and imports extracellular Zn into the cytosol [23,25].

We have shown in this study that Zn administration increases the Zn content in the cells of the small intestine (enterocytes) and concomitantly increases the protein levels of ZnT1, ZnT4, and ZnT5 while reducing ZIP4 once in this tissue. A single administration of Zn can increase Zn exocytosis releasing Zn into the bloodstream via ZnT4/ZnT1 while reducing Zn intake in the intestinal lumen [30]. Thus, Zn administration increases the expression of proteins that protect cells from over accumulating Zn (protective mechanisms). The effect of dietary Zn on the expression of ZnT1 (mRNA and protein) in the rat intestine (duodenum and jejunum) was previously investigated. Diet supplemented with Zn increases intestinal ZnT1 mRNA but not protein [31]. The seeming discrepancy between mRNA and protein expression of ZnT1 could be due to differences in the treatment schedule (acute vs. chronic), the tissues involved (intestinal tissues), and the dose of Zn administered.

Additional administration of IMI enhanced the Zn content in the cells of the small intestine above that elicited by Zn alone. This event was accompanied by a reduction in the Zn-induced increase in ZnT1 and ZnT5 and the normalization of the ZIP4 transporter protein level. Thus, IMI may override the protective mechanisms of cells allowing Zn to be further accumulated.

The next parameter measured in our study was serum Zn levels. We observed increased Zn levels in serum after the administration of Zn alone treatment and lower but still high levels of Zn after Zn + IMI treatment vs. control. Increased serum Zn might reflect the effect of treatment on intestinal Zn permeability as the in vitro experiment demonstrated enhanced permeability of Zn following IMI treatment of colon epithelial Caco-2 cells. A statistically significant effect was observed 30 min after the treatment of Caco-2 cells; this trend was still evident 90 min after the treatment. Our results are consistent with those of Opoka et al. [32], who showed an increase in the efficiency of zinc transport after 30 and 60 min. On the other hand, although serum Zn levels were higher after co-treatment with Zn + IMI, they were much lower when Zn was administered alone. This effect might be due to the increased accumulation of Zn in the intestine induced by combined treatment (reduced ZnT1 activity), leading to the blockade of Zn release in the bloodstream and a concomitant reduction of blood Zn levels. Of course, other mechanisms e.g., redistribution of Zn to organs, might also participate in this process. Although treatment with IMI alone increased serum Zn levels, there were no alterations in levels of Zn transporter proteins, suggesting that other mechanisms may be involved in this phenomenon.

The second part of this study evaluated the brain effects of treatment. Zn or IMI administration did not affect brain Zn levels; however, the combined treatment with both agents increased Zn levels. This effect prompted us to examine selected Zn transporters in the brain with known involvement in mood activity (PFC and Hp). Co-treatment of mice with Zn + IMI increased ZnT4 levels in the PFC and thus supports the increased accumulation of Zn seen in Caco-2 cells. Our previous report indicated some alterations in the levels of Zn transporters. We demonstrated increased levels of ZnT1, ZnT4, and decreased levels of ZnT3 in the PFC in suicide and depression [28]. Similarly, rats subjected to the olfactory bulbectomy (OB) model with respect to ZnT1 and ZnT3 did not reveal any alterations in ZnT4 protein levels [27]. In the present studies, we found decreased levels of ZnT1 in the PFC and ZnT1 and ZnT4 in the Hp after Zn treatment. Zn + IMI co-treatment increased the levels of ZnT4 protein in the PFC and normalized the levels of ZnT1 in the Hp. IMI given alone did not exert any influence on the level of ZnTs in the brain.

**Figure 8:**
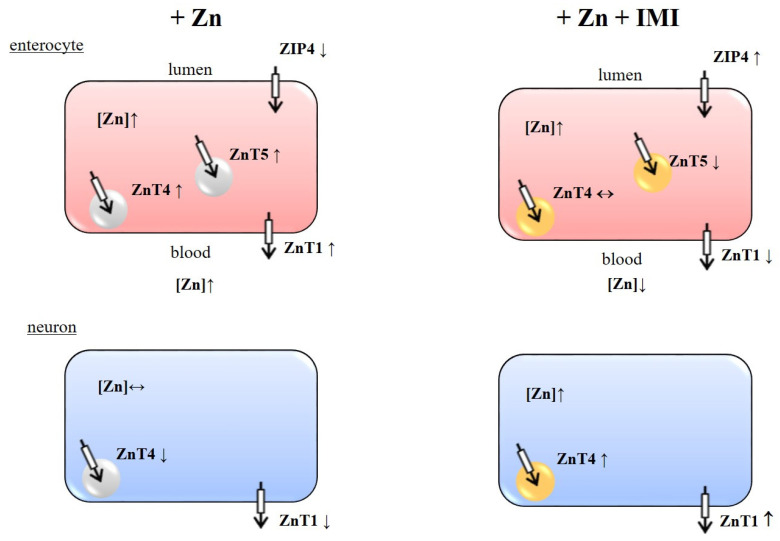
Schema of the effect of Zn (+ Zn) and Zn + IMI (+ Zn + IMI) treatment on zinc transporters in enterocytes and neurons. Symbols: ↑ increase, ↓ decrease, ↔ no alterations. Symbols in figures + Zn + IMI indicate alterations vs. + Zn.

The data presented in this study strongly indicate that IMI enhances Zn absorption in the intestinal tract (presumably involving ZnT1, ZnT4, ZnT5, and ZIP4), influences Zn redistribution between the blood (peripheral organs) and brain, and increases the accumulation of Zn in the intracellular pools (may involve cortical ZnT4) (Figure 8). These mechanisms might be responsible for the beneficial effect of combined IMI/Zn treatment in human depression.

## Figures and Tables

**Table 1:** Effect of treatments on spontaneous locomotor activity in mice.

Treatment (mg/kg)	Distance Travelled (cm)
Control Zn 40 IMI 60 Zn 40 + IMI 60	618.0 ± 72.29 487.2 ± 108.3 466.7 ± 93.61 348.5 ± 58.32

Zn, IMI and NaCl (control) were administered *p.o.* 60 min before the test. Distance travelled was recorded between the 2nd and the 6th min of the test. Each experimental group consisted of 9–12 animals. Data are presented as the means ± S.E.M (two-way ANOVA followed by Tukey *post-hoc* test).

**Table 2:** Effect of IMI on the concentrations of Zn in mouse serum and brain.

Treatment (mg/kg)	Zn Conc. in Serum (µg/mL)	Zn Conc. in Brain (µg/g)
**A.**		
Control	0.4 ± 0.04	12.0 ± 0.19
Zn 40	6.0 ± 0.85 ****	11.6 ± 0.08
Zn 40 + IMI 60	3.8 ± 0.48 **** ^#^	13.1 ± 0.14 **** ^####^
**B.**		
Control	0.6 ± 0.1	16.3 ± 0.35
IMI 60	1.5 ± 0.15 *	16.5 ± 0.35

Zn, IMI and NaCl (control) were administered *p.o.* 60 min before decapitation. Each experimental group consisted of 8–11 animals for A and 3–4 for B. Data are presented as the means ± S.E.M. * *p* < 0.01, **** *p* < 0.0001 vs. control group; ^#^ *p* < 0.05, #### *p* < 0.0001 vs. *Zn* treated group (one-way ANOVA followed by Tukey *post-hoc* test for A; *t*-test for B).
